# Recent Progress of 2D Layered Materials in Water-in-Salt/Deep Eutectic Solvent-Based Liquid Electrolytes for Supercapacitors

**DOI:** 10.3390/nano13071257

**Published:** 2023-04-02

**Authors:** Krishnakumar Melethil, Munusamy Sathish Kumar, Chun-Ming Wu, Hsin-Hui Shen, Balaraman Vedhanarayanan, Tsung-Wu Lin

**Affiliations:** 1 Department of Chemistry, Tunghai University, No.1727, Sec.4, Taiwan Boulevard, Xitun District, Taichung City 40704, Taiwan; 2 National Synchrotron Radiation Research Center, Hsinchu 30076, Taiwan; 3 Department of Materials Science and Engineering, Monash University, Clayton, VIC 3800, Australia; 4 Department of Applied Chemistry and Biotechnology, Graduate School of Engineering, Chiba University, 1-33 Yayoi-cho, Inage-ku, Chiba 263-8522, Japan

**Keywords:** supercapacitors, water-in-salt, deep eutectic solvents, 2D layered materials, electrical double-layer capacitance, pseudocapacitance

## Abstract

Supercapacitors are candidates with the greatest potential for use in sustainable energy resources. Extensive research is being carried out to improve the performances of state-of-art supercapacitors to meet our increased energy demands because of huge technological innovations in various fields. The development of high-performing materials for supercapacitor components such as electrodes, electrolytes, current collectors, and separators is inevitable. To boost research in materials design and production toward supercapacitors, the up-to-date collection of recent advancements is necessary for the benefit of active researchers. This review summarizes the most recent developments of water-in-salt (WIS) and deep eutectic solvents (DES), which are considered significant electrolyte systems to advance the energy density of supercapacitors, with a focus on two-dimensional layered nanomaterials. It provides a comprehensive survey of 2D materials (graphene, MXenes, and transition-metal oxides/dichalcogenides/sulfides) employed in supercapacitors using WIS/DES electrolytes. The synthesis and characterization of various 2D materials along with their electrochemical performances in WIS and DES electrolyte systems are described. In addition, the challenges and opportunities for the next-generation supercapacitor devices are summarily discussed.

## 1. Introduction

Energy consumption based on fossil fuels needs to be replaced with sustainable means to avoid environmental issues as well as cost factors [1,2]. Clean and sustainable energy sources are urgently needed to avert our increasing energy and environmental crises. Electrochemical conversion and energy storage devices are essential parts of the renewable energy cycle and have drawn more attention from researchers, including batteries, fuel cells, and electrochemical capacitors (sometimes known as “Supercapacitors”) [3]. Among them, supercapacitors are widely utilized for electric vehicles, power tools, and portable electronics owing to their higher power density and extremely high cyclic stability compared to their alternatives such as capacitors and batteries [4,5,6]. The electrode materials have a major impact on how well a supercapacitor performs. The development of electrochemical energy storage technologies with greater power/energy density and longer lifetimes is now the main area of research. Advanced 2D nanomaterials must be used in crucial parts, such as anodes, cathodes, and membrane separators, to accomplish the goal [5,6,7].

In general, batteries and supercapacitors show different energy storage mechanisms depending on the materials in the electrodes [8,9,10,11]. For example, the electrical double layer (EDL) resulting from the arrangement of ions at the electrode–electrolyte interface mainly constitutes the charge storage mechanism of supercapacitors. In addition, on the other hand, the oxidation and reduction (redox) reactions of the active materials, due to ion intercalation and deintercalation between the anode and cathode, respectively, are responsible for batteries. Further, in addition to the EDL capacitance, the redox reactions (pseudocapacitive/battery-like behavior) are also responsible for the energy storage mechanism of electrochemical supercapacitors [12]. For example, the energy storage process mainly depends on the oxidation and reduction (redox) reactions of the active materials in batteries due to ion intercalation/deintercalation into/from the anode and cathode, respectively. Further, in terms of electrochemical performance, supercapacitors possess an inferior energy density to that of batteries, which should be addressed to widen the application of the former [13]. Like electrode materials, electrolytes also play a crucial role in improving the overall performance of supercapacitor devices. The electrochemical stability of the electrolyte dominates the operating potential window that eventually determines the device’s energy density [14]. The electrochemical stability window (ESW) is the range of voltage within which the device can function without significantly decomposing the electrolyte at the electrode junction. For the supercapacitor device with an aqueous electrolyte (conventional electrolyte system for supercapacitors), the ESW would normally be limited by the water electrolysis at 1.23 V [15]. However, for water-in-salt (WIS) and deep eutectic solvent (DES) electrolytes, a substantially wider ESW is attained [16,17]. This review summarizes recent advancements in supercapacitor technology, focusing on the use of the above electrolyte systems and the utilization of two-dimensional (2D) layered material-based electrodes.

## 2. Types of Supercapacitors

In the past, conventional dielectric capacitors were a basic component of electrical circuits for storing electrical energy with a capacitance range of microfarads. Later, the invention of the electrical double-layer formation led to the evolution of electrochemical supercapacitors, which can store huge amounts of energy compared to conventional capacitors [18]. Supercapacitors are mainly divided into three types: EDL capacitors, pseudocapacitors, and hybrid supercapacitors based on their energy storage mechanisms. The SCs with EDLC store energy in the form of an electrical double layer through ion adsorption, whereas pseudocapacitors preserve energy by quick surface redox reactions. The hybrid SCs are a combination of EDLC and pseudocapacitors [19].

### 2.1. Supercapacitors with EDLC Behavior

**Figure 1:**
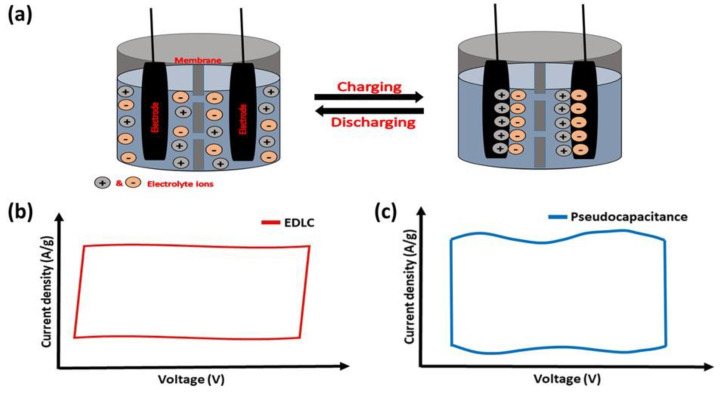
(**a**) Schematic representation showing the energy storage mechanism of EDLC supercapacitors. Representative CVs of (**b**) EDLC and (**c**) pseudocapacitance behaviors.

Among other types of SCs, EDLC SCs are the most used for electric vehicles and the storage of electrical energy generated by different energy resources. In EDLC SCs, both water and organic-based electrolytes have been utilized, and a variety of carbonaceous materials have been employed as electrodes. These include activated carbon, amorphous carbon powder, carbon fibers, carbon nanotubes, carbon-based quantum dots, and aerogels [20]. For EDLC SCs, the energy is mainly stored via non-faradaic processes (electrostatically) [21]. The oppositely charged ions re-orient to generate the Helmholtz double layers at the electrode–electrolyte interface. The potential-dependent surface energy stored at the interface between electrodes generates a double-layer capacitance through electrostatic force (Figure 1a). The nature of CV corresponding to an ideal EDLC behavior that usually exhibits rectangular-shaped curves is shown in Figure 1b. The greater surface area of electrodes and the higher thicknesses of Helmholtz double layers lead to the high capacitance of EDLC SCs. They further possess good durability and cyclic stability over several thousands of charge–discharge cycles [22].

Graphene’s huge surface area and conductivity make it one of the most often used electrode materials for EDLC SCs [23]. The nitrogen doping of graphene materials enhances the electrochemical activity [24]. The crumpled nitrogen-doped graphene, for instance, has a maximum capacitance of >300 F g^−1^ (5 mV s^−1^) in alkaline electrolytes with a BET surface area of 465 m^2^ g^−1^ and pore volume of 3.42 cm^3^ g^−1^ [25]. A comparative study of nanoporous and nonporous carbon electrodes has demonstrated the important effect of nanoporosity in electrode materials for enhancing electrochemical performances [26]. The electrosorption of electrolyte ions over the electrodes is very limited and they interact only at the surface of the nonporous electrode without any transformation in the bulk electrolyte concentration during the charging and discharging. However, the better electrosorption of ions on the porous electrodes improves the power density and lifetime of EDLC SCs. To be more environmentally friendly, carbon electrodes can be prepared from biowaste as the precursor material. For example, the carbon material is synthesized by the pyrolysis of waste banana fibers and subsequently treated with KOH and ZnCl_2_ to create porosity [27]. This process increases the activated carbon electrode surface area by up to 30 times more than that of the untreated material. The resulting carbon electrode shows a specific capacitance of ~70 F g^−1^ (500 mA g^−1^) and better Coulombic efficiency (>85%) with a high current density of over 500 cycles. Moreover, mesoporous carbon materials with cylinder and gyroid nanostructures are synthesized by using amphiphilic polyethylene oxide-block-caprolactone (PEO-PCL) as a template [28]. The prepared cylinder (135 F g^−1^) and gyroid-shaped (155 F g^−1^) carbon materials show better capacitance values in 6 M KOH electrolyte. Furthermore, these two materials show 96 and 105.6 F g^−1^ in 1M tetraethylammonium tetrafluoroborate, respectively [28].

### 2.2. Pseudocapacitance-Based Supercapacitors

The basic mechanism of energy storage in pseudocapacitors involves redox reactions on the electrode surface [29]. In general, pseudocapacitors display 10–100 times higher specific capacitance than EDLC devices because of faradaic charge transfer taking place in the active materials. These types of electrode materials show near-rectangular CVs with broader peaks (for example, as shown in Figure 1c) due to the surface-confined very fast electron transfer reactions. Many transition-metal oxides and conducting polymers exhibit pseudocapacitive properties. The ability of transition-metal oxides to exhibit multiple valence states enables them to exhibit pseudocapacitive behavior [30,31]. In addition, manganese dioxide is one of the best-performing pseudocapacitor active electrodes among various metal oxides and the effect of different phases and morphology of MnO_2_-based electrodes have been extensively reported. For example, MnO_2_ with different phases (α-, β-, and γ-phase) have been prepared through coupled microwave–hydrothermal reaction in the MnCl_2_−KMnO_4_ aqueous solution system. The plate-shaped γ-phase with a trace of β-MnO_2_ shows better performance in capacitance studies [32]. Similarly, ruthenium dioxide (RuO_2_) is a highly conductive metal oxide that has garnered attention for its exceptional electrochemical reversibility, high capacitance, and long lifespan [33]. However, less surface area is considered one of the demerits of ruthenium dioxide. It has been overcome by preparing the different nanostructures of RuO_2_. For instance, the hydrothermally prepared RuO_2_-based porous structures provide a greater surface area of 159.4 m^2^ g^−1^. The RuO_2_ electrode delivers a capacitance of 400 F g^−1^ (0.2 A g^−1^) and maintains a capacitance retention of 84.7% after 6000 GCD cycles (10 A g^−1^) [34]. Moreover, the other metal oxides of copper, cobalt, zinc, titanium, iron, and tungsten also exhibit great performances as active electrode materials in pseudocapacitors [35,36,37,38,39,40]. Metal oxides that include several different metal elements have recently drawn a lot of interest as electrode materials for pseudocapacitors. For example, the NiO and MnO_2_-modified V_2_O_5_ nanoribbon is an example of a ternary metal oxide electrode material. The nanoribbon improves energy (138 Wh kg^−1^) and power (450 W kg^−1^) densities with an extended lifespan (retaining 83.6% after 10,000 cycles), and high capacitance (788 F g^−1^ at 5 mV s^−1^). The above characteristics result from the reactive surfaces with interpenetrating channels of electrode material, which promote effective electron and ion conduction [41].

In the field of electrochemistry, polyaniline has been the focus of substantial research and it is regarded as a useful polymer for supercapacitor applications due to its outstanding electrochemical response, which includes multi-redox behavior, flexibility, and low cost [42,43]. The electrochemical performances of PANI-based electrodes are increased by engineering their morphology, doping with heteroatoms, or forming composites with conductive carbon [44,45,46,47,48]. Likewise, polypyrrole (PPy) and its composites show interesting redox properties as well as good electrical conductivity [49,50,51,52]. However, the practical applications of PANI and PPy polymers are limited by their poor cyclic stability due to the repeated volumetric swelling and shrinkage during GCD experiments. The instability of the conducting polymer has recently been overcome by coating the conducting polymer with thin carbonaceous shells [53].

### 2.3. Hybrid Supercapacitors

**Figure 2:**
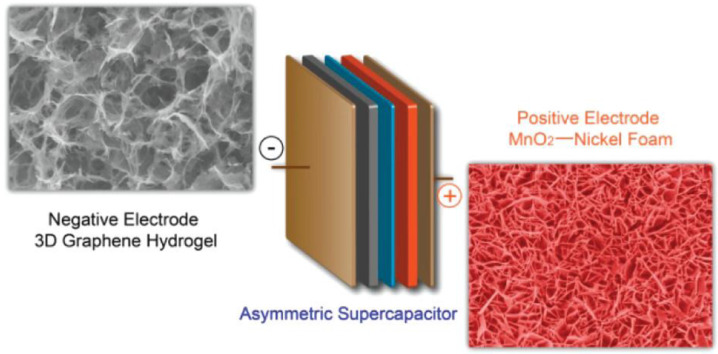
Graphical representation of an HSC device with graphene hydrogel and MnO_2_ nanoflakes. Reprinted with permission from [58].

Hybrid supercapacitors (HSC) provide better energy density, power density, and life span than the individual components [54,55]. HSCs are the combinations of non-faradaic EDLC and faradaic pseudocapacitance [56,57]. In general, a carbon electrode and metal oxide are used as an EDLC and pseudocapacitive electrode in the HSC configuration, respectively. Figure 2 shows the device structure of an HSC made of 3D graphene hydrogel (GH) and the MnO_2_ nanoflakes over a nickel foam as the anode and cathode, respectively. A metal oxide with high intrinsic capacitance provides good energy density, whereas the carbon electrode supplies high power density. As a result, this HSC exhibits a 23.2 Wh kg^−1^ energy density and a 1.0 kW kg^−1^ power density [58].

A hybrid device comprising graphene oxynitride (GON) electrodes exhibits a capacitance of 783.5 F g at a current density of 1 A g^−1^. The GON electrodes are produced over a low-temperature hydrothermal route with an ammonia solution. This preparation method results in the formation of quaternary, pyrrolic, pyridinic, and pyridinic-N-oxides on graphene oxide (GO), which not only improves the capacitance owing to their positive charges but also enhances the electron transfer within the GO. A GON-based HSC exhibits a good cyclability of 5000 cycles with an 87% efficiency in PVA/KOH gel electrolytes and produces a 40 Wh kg^−1^ energy density at a 900 W kg^−1^ power density [59].

The electrode in the HSC can also exhibit battery-like behavior where a pair of redox peaks is usually observed in its CV curve. The redox reaction in this instance takes place in the electrode and is kinetically regulated by diffusion. For example, Co_3_O_4_-decorated P, N-co-doped porous carbon material (PNC) is one of the representative examples of battery-like electrodes. When the electrode is combined with activated carbon (anode), the resulting HSC with a 6 M KOH electrolyte demonstrates better energy density (47.18 Wh g^−1^), power density (375 W kg^−1^), and possesses a capacitance retention of 92% (5000 GCD cycles). The electrochemical performance is improved because of the higher surface area resulting from the porous structures of PNC and the small particle size of cobalt oxide. Furthermore, the strong anchoring effect of PNC on Co_3_O_4_ nanoparticles, along with the confinement effect of the nanocavities, helps to maintain the stability of the supercapacitor [60]. Similarly, the flower-like mesoporous NiCo_2_O_4_ structures synthesized using the solvothermal method can serve as a battery-type behavior that exhibits a 122.5 C g^−1^ specific capacity at 1 A g^−1^ in a 6M KOH electrolyte. This electrode maintains decent cycling stability, with a loss of just 21% in specific capacity over 6000 cycles at 2 A/g, and demonstrates an improved electrochemical performance with an increase in the concentration of aqueous electrolyte [61].

## 3. Unconventional Aqueous Electrolytes

Supercapacitors commonly use aqueous, solid, gel, organic, ionic liquid, and redox-based electrolytes [62,63,64,65,66,67]. Among liquid electrolytes, aqueous electrolytes are an all-time favorite of supercapacitor research because of their higher ionic conductivity, great safety, and low cost. However, the low operation voltage due to the decomposition voltage of water (at 1.23 V) restricts the energy density [68]. In contrast, the operating potential window of organic electrolytes can achieve up to 4 V. The drawbacks of organic electrolytes are their high volatility, flammability, and high resistance [69]. In the case of ionic liquids, their disadvantages such as toxicity, difficulty in preparation, and high-cost limit their practical application in supercapacitors [70]. As a result, the search for alternative electrolytes that not only maintain all of the benefits of conventional electrolytes but also offer a wider voltage window, environmental friendliness, and good conductivity is highly demanded [71,72]. The water-in-salt (WIS) electrolyte is a rising star in the electrolyte field because it inhibits the water-splitting reaction and shows a larger ESW value. Because the specific energy (E = CV^2^/2) highly depends on the specific capacitance (C) and operating voltage (V) of supercapacitors, the expanded ESW of the electrolyte reflects the reinforcement of the operating voltage [73]. Moreover, the development of solid electrolyte interphase (SEI) is seen as a benefit of the WIS electrolyte [74]. The presence of SEI further widens the ESW by suppressing the hydrogen evolution reaction (HER) at the electrode interface [75].

### 3.1. Water-in-Salt Electrolytes

**Figure 3:**
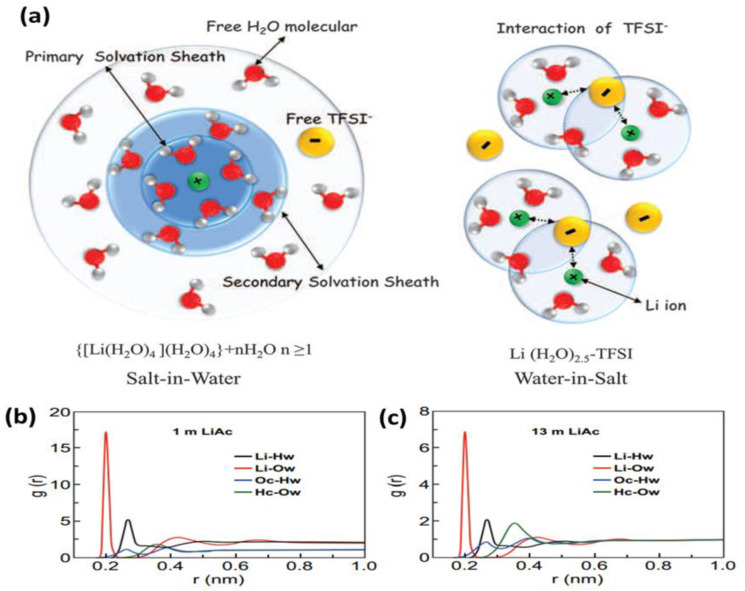
(**a**) A pictorial comparison of the structures of salt-in-water and WIS electrolytes. Reprinted with the permission of [72]. RDF curves of (**b**) 1 m LiAc and (**c**) 13 m LiAc electrolytes obtained using molecular dynamics (MD) simulation. Reprinted with the permission of [82].

The salt-in-water (SiW) electrolytes are the most used aqueous electrolyte in supercapacitors. In SiW, the amount of solute is less than the solvent (water) concentration. However, in recent years, researchers have started to make higher concentration water-in-salt electrolytes (WIS). A WIS electrolyte is a solution with a high concentration of salt in which the solute has a higher mass or volumetric content than the solvent (water) [76]. Figure 3a perfectly pictures the arrangements and interaction of water and lithium bis(trifluoromethane sulfonyl) imide (LiTFSI) in the SiW and WIS systems [72]. The excess number of free water molecules causes a hydrogen evolution reaction (HER) in SiW. This side reaction decreases the efficiency of the supercapacitor by limiting the ESW. The lack of interaction between cation and anion is also observed in SiW because of the low solute ion availability in a specific area of the system. On the other hand, the water clusters present in the WIS system are completely engaged with salt ions. This situation blocks the HER reaction and provides a higher ESW. Moreover, the WIS electrolyte’s high solute ion density per surface area improves the interaction between solute ions. Because of these qualities, the water-in-salt solution is thought to be a viable nonconventional electrolyte for supercapacitors.

The aqueous LiTFSI electrolyte was made by Wang and colleagues in 2015, and it had a concentration of 21 (m), which expanded the ESW value to ~3 V. The cell constructed with LiMn_2_O_4_ as a cathode, Mo_6_S_8_ as an anode, and 21 m LiTFSI electrolyte possesses a discharge capacity of 47 mAh g^−1^ with an energy density of 84 Wh kg^−1^ [72]. Furthermore, the battery maintains good cycle stability with a capacity retention of 68% at a high rate of 4.5 C and shows nearly 100 % Coulombic efficiency after 1000 cycles. Other WIS electrolyte systems include high-concentration solutions of sodium perchlorate, ammonium acetate, potassium acetate, sodium acetate, and sodium nitrate [77,78]. The studies of the abovementioned electrolytes have demonstrated that the voltage window of the SC increases when the electrolyte transforms from salt-in-water to water-in-salt. The maximum voltage window obtained from different WIS systems is 2.7 for 17 m NaClO_4_, 2.3 for 11 m NaNO_3_, 1.9 for 7 m CH_3_COONa, and 2.0 V for 27 m CH_3_COOK. This result may be attributed to the interaction between water molecules and the anions. Generally, the anions in WIS that dissociate hydrogen-bonded water clusters result in a better cell voltage. The NO^3−^ and ClO^4−^ anions tear down the water framework, whereas the acetate (CH_3_COO^−^) anions build it up. According to the above studies, NO_3_^−^ and ClO_4_^−^ ions can provide a greater cell voltage than acetate anions [79]. Similarly, cation size is an important factor in WIS electrolytes. In the case of acetate-based WIS electrolytes, the potassium ions exhibit better charge storage characteristics than ammonium ions because the former shows superior diffusion and intercalation power to the latter [80]. Furthermore, ambipolar WIS redox electrolytes receive more concern due to their excellent pseudocapacitance. The micro-supercapacitor (MSC), composed of exfoliated graphene (EG) nanosheet electrodes (as both cathode and anode) with 15 m zinc chloride (ZnCl_2_) and 1 m zinc iodide (ZnI_2_), formed I_2_ at the EG-electrolyte interphase during charging by the oxidation of I^−^ ions. Thus, I_2_ is gradually proportionate with I, I_3_, and then I_5_ as its quantity rises. The utilization of I^−^ with increasing voltage navigates the dismutation of I_3_^−^ and I_5_^−^. Finally, the [ZnI_x_(OH_2_)_4−x_]^2−x^ species also start to oxidize to I_2_ as a complimentary iodide source. The above-discussed reactions create a shortage of I^−^ in electrolytes and this shortage suppresses the formation of extra polyiodides that can shuttle into the electrolyte. In the discharging process, the reduction of I_2_ releases more I^−^ ions, and I^−^ ions react with the remaining I_2_ to form I_3_^–^ followed by I_5_^−^. Finally, these iodide ions coordinate to [ZnI_x_(OH_2_)_4−x_]^2−x^ species of the electrolyte. This coordination complex-forming reaction enhances the Coulombic efficiency and stability by suppressing the formation of polyiodide (I_3_^−^ and I_5_^−^) and the subsequent dissolution of polyiodide ions to the electrolyte. Moreover, the interaction of Zn^2+^ with water molecules lowers the freezing point. Thus, the ZnCl_2_–ZnI_2_ WIS electrolyte provides enhanced capacity, even at −20 °C [81]. The WIS electrolytes work well with battery-type electrode materials and exhibit better electrochemical performances. For example, the organic polymer composed of mixed aromatic amine groups exhibits battery-type behavior in the 30 m ammonium acetate WIS electrolyte [78]. The granular-like morphology of self-assembled polymeric chains is more suitable for improved electrolyte intercalation into the polymeric structures. The CV and GCD plots show the well-separated redox peaks (due to the reversible conversion of aromatic benzenoid forms to quinoid forms) and potential plateau, respectively, which are evident in the battery-type behavior. The above polymer is serving as a cathode material for a battery–supercapacitor hybrid device with better electrochemical performance.

In addition, the high concentration of salt in WIS increases the interaction between water molecules and salts. This result is confirmed by the radial distribution function (RDF) studies of lithium acetate (LiAc) electrolytes. The RDF curve of Figure 3b,c implies that the distance between the H atom linked with the C atom in the acetate anion (Hc) and the O atom in H_2_O (Ow) fluctuates between 0.36 (1 m LiAc) and 0.35 nm (13 m LiAc). This indicates that the interaction between H_2_O molecules and acetate ions develops strongly in concentrated electrolytes [82].

### 3.2. Deep Eutectic Solvents Electrolytes

The deep eutectic solvent (DES) is an example of a green electrolyte formed by the association of two or three components with self-stabilization through hydrogen bonding. This solvent possesses nonflammability, compositional tunability, low vapor pressure, and good thermal and chemical stability. It attracts special interest due to the ease of preparation and low production cost. In addition to acting as an electrolyte, DESs have also been utilized as sources of carbon/metal, templates, and active reagents in the production of nanomaterials [70]. DESs can be used in many different applications, including electrochemistry, material chemistry, catalysis, metal processing, separation, and extraction [83,84,85,86,87]. The DES is a multicomponent system in which the correct acceptors and donors for hydrogen bonds come together to form a complex. There are four different types of DES, each determined by the combination of quaternary ammonium salts with different substances. Type I consists of quaternary ammonium salts united with metal chloride, type II is comprised metal chloride hydrate and ammonium salts, type III is a combination of hydrogen bond donors with ammonium salts, and type IV is a combination of metal salts and hydrogen bond donors [88].

**Figure 4:**
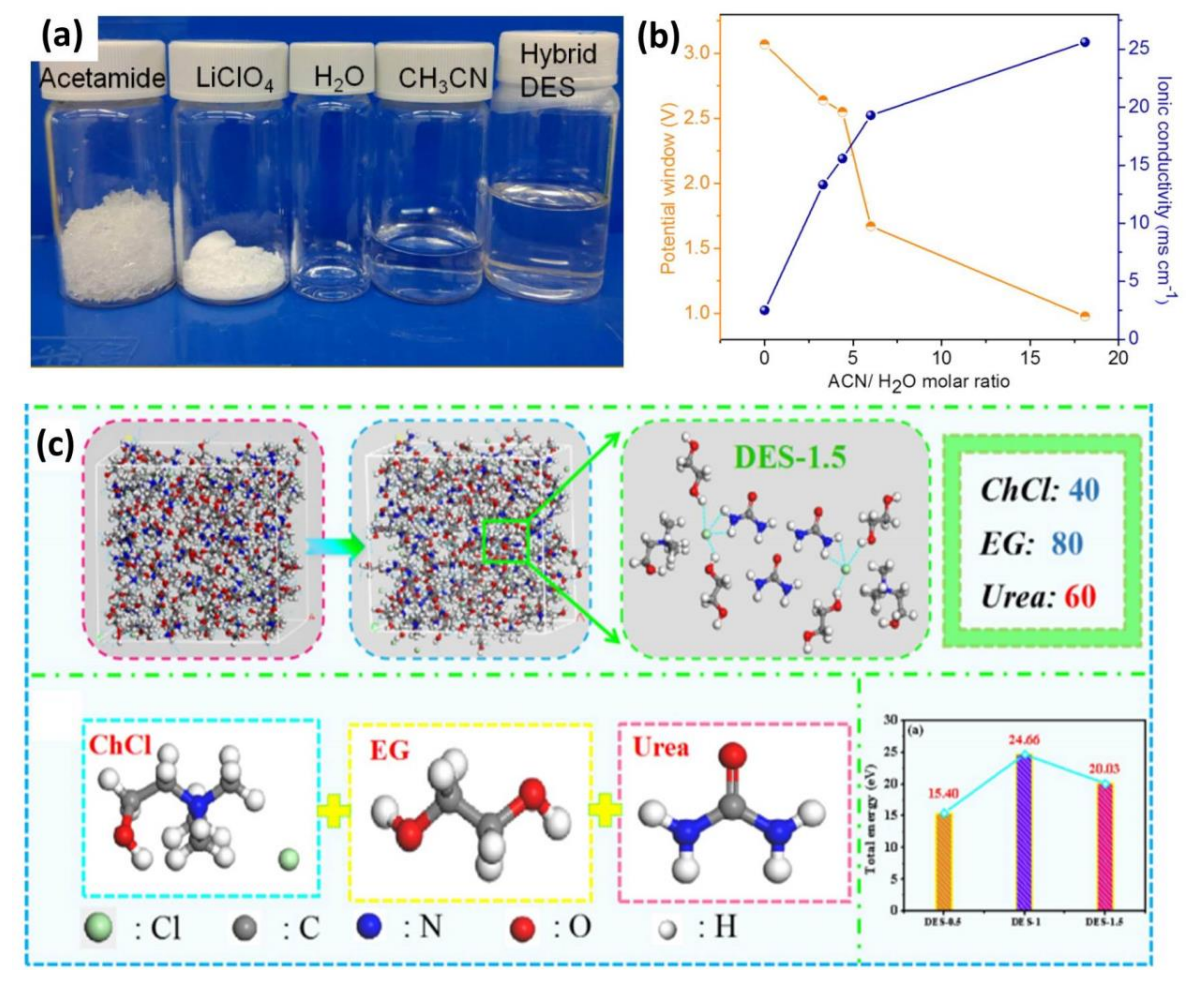
(**a**) Photograph of the corresponding components of a hybrid DES. (**b**) A plot illustrating the changes in ionic conductivity of DES electrolytes with varying molar ratios of acetonitrile/water mixtures. Reprinted with the permission of [90]. (**c**) MD simulation of a DES system prepared from the ternary mixture of choline chloride, ethylene glycol, and urea. Reprinted with the permission of [91].

The formation of DES will take place only when intermolecular interactions between various components are greater than the original effects in each component. The noncovalent interactions such as hydrogen bonding, van der Waals force of attraction, and Lewis acid−base interactions are present in DES. These interactions determine the surface tension, ionic conductivity, melting point, and viscosity of the formed DES [89]. The high viscosity of a DES has been considered one of the drawbacks; however, the viscosity can be reduced by the addition of water as a co-solvent, referred to as a hydrated DES. The acetonitrile and water together in mixing with the acetamide-based DES electrolyte is a good example of improving the conductivity and viscosity of the DES electrolyte (Figure 4a,b). Moreover, the co-solvent addition (water and acetonitrile) enhances the flame retardant properties, antifreezing properties, and ionic conductivity of hybrid DES electrolytes [90].

A ternary DES electrolyte consists of three different components with a specific molarity ratio. One of the instances is combining choline chloride, ethylene glycol, and urea in a mixture with a (1:2:1) molar ratio [91]. This ternary DES electrolyte shows a supramolecular nature by forming an H–H interaction between ethylene glycol and urea. This interaction has been confirmed by FT-IR and DFT studies. In the DES system, the combination of these three elements results in a broad and strong absorption peak in the range of 3250 cm^−1^, indicating the formation of hydrogen bonds between ChCl, urea, and ethylene glycol. DFT simulation calculations typically show the system’s structure as well as various parameters such as total energy and the diffusion coefficient. Figure 4c, from DFT studies, illustrates the establishment of a hydrogen bond among the Cl atoms, H atoms, and -OH atoms of the DES components. At various temperatures, this hydrogen bond generation supports the stability of electrolytes. Furthermore, the oxygen-present functional groups (-COOH and -OH) at the surface of the activated carbon electrode can interact with the free hydroxyl ions to generate additional molecular hydrogen bonds and speed up the migration of electrolyte ions. In addition, DES prepared using lithium bis((trifluoromethyl)sulfonyl) imide (LiTFSI) combined with ethylene glycol (EG) diluted by either ethylene carbonate (EC) or acetonitrile (ACN) produces a broad peak in FT-IR spectra due to the vibration of OH bonds at 3313 cm^−1^ [92]. This peak provides clear evidence of new H–H bond formation.

DES electrolytes offer advantages over other types of electrolytes, such as overcoming the harmfulness and protection concerns associated with organic-based electrolytes, reducing the high price of ionic liquid-based solvents, and increasing the inadequate electrochemical stability window of aqueous electrolytes [92]. As a result, they have drawn a lot of interest and are now the current topic of energy research. For example, the DES system is prepared by lithium salt using LiNO_3_, LiPF_6_, or LiTFSI, and the N-methyl acetamide (MAc) is used as the electrolyte for the SC with an activated carbon electrode. The LiTFSI−MAc and LiNO_3_−MAc DES electrolytes have a good conductivity of 10 mS/cm and a low viscosity of 12 mPa·s at elevated temperatures [71]. This device can be safely operated up to 2.8 V in the temperature range of 25 °C to 80 °C. Nevertheless, the electrical conductivity and fluidity of the DES electrolyte are not favorable at ambient temperatures when compared to that at 60–80 °C. Hence, the device with the above DES electrolyte has a preferred operation temperature of around 80 °C for higher electrochemical performance.

## 4. Supercapacitor Performance of Two-Dimensional (2D) Electrode Material in Novel Aqueous Electrolytes

Two-dimensional materials are promising electrodes for supercapacitors because of their excellent capacitive performances, high energy, long cycle life, and power density. MXenes, graphene materials, metal oxides, metal nitrides, metal sulfides, and metal–organic frameworks are some examples of 2D materials [93]. The porous structure, surface area, and capability for ion intercalation are some factors contributing to the improved performance of 2D electrode materials. Recently, supercapacitors made from the combination of 2D electrode materials with nonconventional electrolytes have attracted special concern due to their high performance. Table 1 compares the electrochemical performances of a few 2D layered materials with different WIS/DES electrolyte systems.

### 4.1. Graphene and Graphene Derivatives

Carbon atoms are arranged in a hexagonal honeycomb lattice to form the 2D nanomaterial known as graphene. It is considered an interesting and favorite material for supercapacitors due to its outstanding electrical conductivity, huge surface area, high thermal conductivity, and mechanical properties [108]. In general, graphene can be synthesized through either a top-down or bottom-up process. The top-down method involves the physical or chemical exfoliation of bulk graphite, which reduces the thickness of graphene to a single layer [109,110,111,112,113]. For example, the graphene oxide (GO) prepared by the oxidation of graphite through Hammer’s method is the typical product obtained from the top-down process. Although the synthesis of GO is a highly preferred technique due to its ease and scalability, the structure of the graphene lattice is significantly damaged after severe oxidation treatment. To partially restore the physical properties of graphene, GO nanosheets are usually subject to a chemical or thermal reduction process. The resulting product is the so-called “reduced graphene oxide” (rGO), which shows the structural transition between perfect graphene and GO [114,115]. On the other hand, chemical vapor deposition (CVD) is viewed as employing the bottom-up technique for graphene synthesis. Large-area graphene can be grown on the surface of metallic catalysts such as copper and nickel substrates [116]. Furthermore, many parameters such as growth temperature, reaction time, carbon source, catalytic substrate, etc., can be used to engineer the interlayer distance and the degree of defects [117].

The active electrodes in batteries, fuel cells, and supercapacitors have frequently been made using materials based on graphene [118]. Graphene-based electrodes and WIS electrolytes have been combined in some SC investigations. For instance, in a NaClO_4_ WIS electrolyte (17 m), the rGO electrode has a capacity of 59.7 F g^−1^ at 0.1 A g^−1^ [95]. The device exhibits an operating window of 2.3 V and possesses 84% (10,000 cycles) of capacitance retention. By adding heteroatoms to the graphene layers’ basal plane, the electrochemical performance of the rGO can be further enhanced. In 17 m NaClO_4_, for example, the nitrogen-doped (N-doped) rGO electrodes show a 140 Wh kg^−1^ energy density at a 640 W/kg power density, with a maximum cell voltage of 2.7 V [79]. Moreover, the electrolyte for rGO-based SCs has been evaluated as 11 M NaNO_3_ WIS [96]. In 11 M NaNO_3_ WIS, the rGO-SC exhibits an operating potential of 2.1 V and a capacitance of 149.4 F g^−1^ at 0.2 A g^−1^. It still possesses the capacitance retention of 98.1% (5000 GCD cycles), and, further, it has a considerable energy density of 22.87 Wh kg^−1^ at 210 W kg^−1^. 

### 4.2. Metal Oxides and Sulfides

Metal oxide or sulfide-based electrode materials have been widely employed in cost-effective and high-performance aqueous pseudocapacitors. Recently, the construction of SCs was demonstrated by using MnO_2_ (positive electrode) and Fe_3_O_4_ (negative electrode)–based electrode materials [97]. The SC with a WIS LiTFSI electrolyte (21 m) offers an energy density of 35 Wh kg^−1^ and a power density of 151 W kg^−1^. This device operates at 2.2 V and has a capacitance retention of 87% (3000 cycles). Similarly, the solid-state reaction produced double metal oxides of niobium and tungsten (Nb_18_W_16_O_93_), which demonstrate a capacity of ~54 mAh g^−1^ and preserve 85% of their initial capacity after 50,000 charge and discharge cycles in 13 m LiAc electrolyte [82].

An advanced hybrid SC with an operating window of 1.5 V is constructed from the MnO_2_, water-stable multilayered lithium, and 21 m LiTFSI functioning as the positive electrode, negative electrode, and WIS as an electrolyte [98]. In general, this hybrid device with the WIS electrolyte exhibits higher energy and power density values when compared to its counterparts having non-WIS-based electrolytes. For example, the hybrid device with 21 m LiTFSI delivers a maximum energy density of >400 Wh kg^−1^_MnO2_ at a power density of 0.88 kW/kg_MnO2_, which shows a higher energy density at the same current density than that of 1 M Li_2_SO_4_. Furthermore, the hybrid SC with a MnO_2_ positive electrode exhibits great energy-retaining capabilities of around 90% within 3000 cycles.

In addition to the transition-metal oxide-containing electrode materials, the transition-metal dichalcogenides TMDs (MX_2_; X = S or Se) have also been employed as active materials [119]. For TMDs, the energy storage mechanism involves both the faradaic and non-faradaic processes due to their characteristics of redox reactions and the enhanced contribution of EDLC from the high surface area [120]. Among the different phases of MoS_2_, the 1T phase has better electrochemical characteristics due to its increased interlayer spacing of 6.15 nm and higher electrical conductivity. This interlayer spacing increased to 7.2 nm by making a composite with rGO, and this enhancement also helps to intercalate more electrolyte ions [90]. The porous nature of the rGO hydrogel and the high electrical conductivity of 1T-MoS_2_ are inherited by the hydrogel created from these two materials, which results in a good performance. This composite hydrogel shows great compatibility with hybrid DES electrolytes. The equivalent symmetric device has a wide operating voltage range of 2.3 V and can run at a maximum power density of 1164 W kg^−1^ with a maximum energy density of 31.2 Wh kg^−1^. Additionally, this device displays remarkable durability, with a capacitance retention of >90% (20,000 cycles).

**Figure 5:**
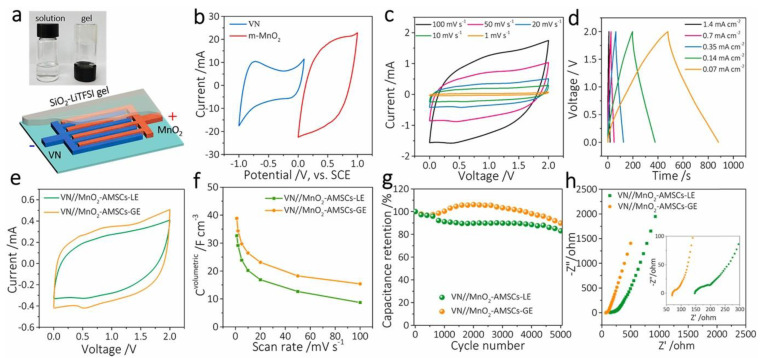
(**a**) A WIS gel electrolyte is shown schematically to be employed in VN/MnO_2_–AMSCs (insets showing the SiO_2_–LiTFSI-based gel electrolyte and 5 M liquid electrolyte of LiTFSI on the left). CV curves of (**b**) m-MnO_2_ and VN electrode materials and (**c**) the fabricated device. (**d**) GCD profiles and (**e**) CV curves of the device with the gel electrolyte. (**f**) Plots of capacitance vs. scan rate, (**g**) the cycling stability, and (**h**) Nyquist plots of the VN//MnO_2_ device with gel and liquid electrolytes, respectively. Reprinted with the permission of [104].

The use of 2D nanosheets as electrodes offers a crucial material platform with significant improvements due to their unique features and the performance of planar micro-supercapacitors (MSCs), including an interlinked porous network and a high specific surface area. For instance, an asymmetric micro-supercapacitor is fabricated using a MnO_2_ nanosheet as a positive electrode and vanadium oxide nanosheets (VN) as the negative electrode in WIS solution (5 M LiTFSI) or WIS gel electrolytes (SiO_2_-LiTFSI), as depicted in Figure 5a,b [104]. According to CV and GCD curves (Figure 5c,d), this asymmetric cell provides a high operating voltage of 2 V and pseudocapacitive behavior. Compared with WIS electrolytes, the WIS gel electrolyte provides a better rate capability, cycling stability, and energy density (Figure 5e–g). For example, the VN//MnO_2_–AMSCs–GE (21.6 mWh/cm^3^) shows higher volumetric energy density than VN//MnO_2_–AMSCs–LE (19.6 mWh/cm^3^). Moreover, the gel electrolyte used in AMSC possesses greater capacitance retention, with 90% retention after 5000 charge–discharge cycles, compared to the liquid electrolyte that AMSC works with, which has 83% retention. (Figure 5g). According to their EIS spectra (Figure 5h), VN/MnO_2_–AMSC with a liquid electrolyte has a lesser charge transfer resistance than that of the device with a gel electrolyte. The better electrode wettability, as well as the improved charge transfer kinetics, account for the enhancement in device performance.

### 4.3. MXenes

The 2D transition-metal compounds known as MXene have the generic chemical formula Mn^+1^X_n_T_x_, (M = transition metal, X = carbon/nitrogen, and T = surface groups (–OH, –F, –O, etc.) [121]. It is a good current carrier due to its metallic nature and better charge transfer characteristics. The transition metal ‘M’ of MXene possesses variable oxidation numbers and some of the MXenes exhibit semiconducting behavior. MXenes have shown great performances as high-rate electrodes for pseudocapacitors due to their highly reversible redox reactions and electrical conductivity. Generally, the MXenes are synthesized using (i) top-down and (ii) bottom-up methods. In the top-down approach, the methods are an etching process by HF acid/fluoride salt, alkalis, molten salt, electrochemical, thermal reduction, halogen/interhalogen compounds, or algae extraction [122]. Chemical vapor deposition (CVD), using templates, pulsed laser deposition (PLD), and sputtering are the main bottom-up methods for MXene synthesis. The main problem of the MXene electrode is the narrow working potential window (≤0.6 V) owing to the oxidation at the high anodic potential in aqueous electrolytes. However, the MXenes show better stability in WIS electrolytes due to the high content of WIS that regulates the anion intercalation at the electrode of MXene. This process efficiently enhances the potential window of MXene-based SCs. In a study, the 2D Ti_3_C_2_T_x_ MXene electrode in 20 m LiCl exhibits an operating window of 1.6 V. The high concentration of lithium chloride not only inhibits the oxidation of MXene but also broadens the ESW. Moreover, the ability of MXene electrode layers to reverse ion intercalation/extraction in high-concentration electrolytes provides better cyclic stability. It possesses a volumetric capacitance of 89.2 Fcm^3^ at 0.1 mA cm^−2^ and an energy density of 31 mWh cm^−3^ at a power density of 250 mW cm^−3^ [99]. The fabrication of a full cell with Ti_3_C_2_ (negative) and MnO (positive) electrodes in 21 m potassium acetate is a solution for the limited cell voltage of MXene-based SCs [94]. The device with a potential window of 2.2V shows a capacitance of 25 F cm^−3^ at 5 mV s^−1^ and a good capacitance retention of 93% after 10,000 cycles. The wavy-layered Ti_3_C_2_T_x_-based electrode material is tested in a 19.2 m LiBr WIS electrolyte, which shows a capacitance of 28 F g^−1^, even at a 10,000 mV s^−1^ scan rate [101]. Although a wavy Ti_3_C_2_T_x_ electrode exhibits a clear pair of CV peaks in the WIS electrolyte, its electrochemical kinetics exhibit less diffusion-controlled and more surface-controlled behavior. The fast-charge storage process results from the unique desolvation-free Li-ion insertion among the layers of wavy Ti_3_C_2_T_x_. The phenomenon of desolvation-free cation insertion has been proved by the electrochemical quartz crystal microbalance (EQCM) experiment and density functional theory (DFT) calculation. These data demonstrate that Li ions with 2.85 coordinated water molecules are intercalated into the layer of Ti_3_C_2_T_x_ at the potential of 0.17 V in the WIS electrolyte, which further leads to the expansion of interlayer distance. Due to the effect of desolvation-free ion insertion, the symmetric cell consisting of Ti_3_C_2_T_x_ and LiCl (~20 m) electrolyte possesses a large operating window of 1.6 V and delivers a cell capacitance of 26 F/g at 2 mV/s.

### 4.4. Two-Dimensional Composite Materials

Two-dimensional composite electrode materials mostly possess improved electrochemical performances over their parent materials and show enhanced specific capacitance, upright energy, power densities, excellent capacitive properties, and long charging/discharging cycles [93]. Composite electrodes play a vital role in shortening the difference in energy density between SC and batteries. The unconventional electrolytes provide a better ESW for these electrode materials. For example, the WIS electrolyte prepared by 32 m ammonium acetate possesses a high conductivity of 23.3 mS/cm and an ESW of 2.13 V [102]. Moreover, it works well with anode electrode materials made of reduced graphene oxide (rGO) and perylene tetracarboxylic diimide (PTCDI). This anode electrode delivers a 165 mAh g^−1^ maximum capacity at 0.5 A g^−1^ by quick ammonium ion intercalation through redox reactions including reversible carbonyl group enolization in PTCDI during charge–discharge processes. The pristine PTCDI electrode shows less capacity than the PTCDI/rGO composite, and the composite electrode gradually increases specific capacity with the higher rGO loading. This result can be attributed to the introduction of rGO, which significantly improves the composite electrode’s electrical conductivity. The HSC made up of activated carbon (cathode), PTCDI/rGO (anode), and 32 m ammonium acetate electrolyte shows a capacity retention of 74% at 1 A g^−1^ and gives a maximum energy density of 12.9 Wh kg^−1^ at the power density of 827 W kg^−1^. Similarly, the rGO–PANI-based composite materials show enhanced electrochemical performance in a hybrid DES electrolyte. The prepared DES electrolyte consists of N-methyl acetamide and lithium perchlorate and its physical properties such as ionic conductivity are supplementarily engineered by the accumulation of co-solvents consisting of water as well as N, N-dimethylformamide (DMF). The hybrid SC, with an operating window of 2.2 V at a power density of ~1 kW kg^−1^, demonstrates a higher energy density of 28 Wh kg^−1^ [103].

**Figure 6:**
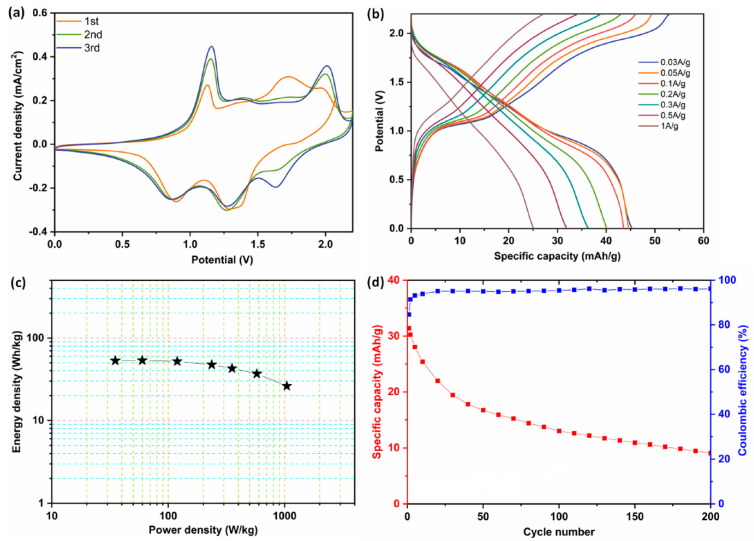
(**a**) CV analysis, (**b**) GCD curves, (**c**) energy density vs. power density plot, and (**d**) cyclic stability of two-electrode systems using PTCDI/rGO (anode), LMO (cathode), and the hybrid Li/Mg electrolyte. Reprinted with the permission of [107].

A novel DES electrolyte composed of magnesium chloride, urea, lithium perchlorate, and water has been created for battery–supercapacitor hybrid systems [107]. Figure 6a displays the CV curves of two-electrode systems with a wide operating voltage window. However, as compared to the first cycle, the CV peaks in the second and third cycles are stronger, demonstrating that events occurring across the cycling process do have an impact on the electrode material’s reactivity. Clear oxidative peaks were observed because of the Li^+^ ion deintercalation from the positive electrode, also with Mg^2+^ ion intercalation into the PTCDI/rGO electrode (Figure 6a). The reductive peaks, on the other hand, are visible at 0.86, 1.27, and 1.63 V, and are caused by the Mg^2+^ ions removal from the negative electrode and the Li^+^ ions insertion into the positive electrode. The GCD investigations at different current densities are depicted in Figure 6b, and they reveal that this two-electrode device can give 43 mAh g^−1^ of capacity achieved at 0.03 A g^−1^ current density. Additionally, at 1 A g^−1^, 55.5% of the overall specific capacity is still present, demonstrating a high-rate capability. Figure 6c shows a Ragone plot (the specific energy of a device is traced against its specific power, which is mainly used to compare the energy and power densities of various devices) of the PTCDI/rGO//LMO full cell with the hybrid Li/Mg electrolyte. The device produces a higher energy density and power density (Figure 6c) with reasonable capacity retention and Coulombic efficiency (Figure 6d).

## 5. Conclusions and Future Perspectives

The faster depletion of fossil fuels and their environmental impact has prompted the research community to seek alternate and viable sources of energy. Electrochemical SCs have emerged as viable energy storage devices due to their excellent charge storage characteristics. In this review, we have discussed various 2D electrode materials and their electrochemical performances in the WIS and DES-based electrolytes by highlighting the advantages and downsides of the supercapacitors. Two-dimensional layered materials have demonstrated superior electrochemical properties, such as high energy and power density, ultra-cyclic stability, and outstanding rate capabilities. Varieties of 2D materials, including graphene, MXene, metal oxides, TMDs, and their composite materials, are covered in this review. Different WIS and DES electrolyte systems have been employed for these layered materials in supercapacitor applications. LiTFSI, LiClO_4_, NaClO_4_, LiCl, CH_3_COONa, and CH_3_COONH_4_ are a few well-known WIS electrolytes that are superconcentrated aqueous solutions. The presence of extremely high concentrations of cations and anions significantly reduces the number of free water molecules, which greatly restricts parasitic side reactions. Multiple interactions between ions and water weaken the intermolecular hydrogen bonds among water molecules and reduce the number of water clusters (H-bonded units). As a result, the ESWs of these WIS electrolytes are notably improved. Further, since the electrolytes consist of a variety of H-bonding interactions between the water and the electrolyte ions, they construct extended network-like structures. In the case of DES, its ESW would not be greatly changed by the addition of a small amount of water or other organic solvents; however, its ionic conductivity would be significantly enhanced. For this, a few organic carbonates (dimethyl-carbonate, ethyl-carbonate, and propylene-carbonate), as well as polar aprotic solvents (acetonitrile and dimethyl formamide), have been employed. Most of these electrolytes have remarkably improved the energy density and cyclic stability of SC devices.

The tuning of the electrode and electrolyte interface is critical for optimizing the performance of supercapacitors, particularly in terms of capacitance and rate capability. The important criteria for advanced applications, such as higher specific energy, power, and ultra-long cycle life, must be taken into account while choosing the right combination of materials. In addition, the development of hybrid battery/supercapacitor systems is a new area of opportunity. Such systems will be highly sought-after in situations where a battery or supercapacitor alone is unable to satisfy certain specifications such as cycle life, energy density, and power rating. The processing of electrodes and cell assembly are equally crucial in the development of unprecedented materials, and they must be carefully adjusted to ensure maximum improvements. In addition, while selecting the electrolyte and electrode materials, it is important to consider a range of factors, such as the temperature working range, self-discharge rate, lifespan, and operational potential, which might also affect the performances of current collectors, separators, and other components of the device. During the design of porous materials as supercapacitor electrodes, it is important to consider the compatibility of the pore size with the electrolyte, as this can significantly impact the performance and stability of the device. This review serves as a valuable resource for researchers seeking to explore the trends in 2D electrode materials and WIS/DES electrolytes in supercapacitors. By gaining insights from this review, researchers can develop new and highly efficient energy storage devices to fulfill the rising demand for sustainable energy solutions globally.

## Figures and Tables

**Table 1:** A summary of the performance of 2D electrode materials in nonconventional electrolytes.

Materials	Electrolyte	Specific Capacitance	Energy Density	Power Density	Cyclic Stability	Ref.
graphene nanoplatelets-coated carbon paper	20 M 1-butyl-3-methylimidazolium chloride + 0.1 M 4-hydroxy-2,2,6,6-tetramethyl piperidin-1-oxyl	480 F g^−1^ at 20 mV s^−1^	110 Wh kg^−1^	10 kW kg^−1^	2.56% capacitance loss after 500 cycles	[94]
reduced graphene oxide and graphene oxide	17 m NaClO_4_	59.7 F g^−1^	43.8 Wh kg^−1^	115.6 W kg^−1^	84% after 10,000 cycles	[95]
N-doped RGO	17 m NaClO_4_	138 F g^−1^ at 1 A g^−1^	140 Wh kg^−1^	10,125 W kg^−1^	98% after 10,000 cycles	[79]
rGO	11 M NaNO_3_	149.4 F g^−1^	22.87 Wh kg^−1^	210 W kg^−1^	98.1% after 5000 cycles	[96]
MnO_2_ (cathode) and Fe_3_O_4_ (anode)	21 m LiTFSI		35.5 Wh kg^−1^	2692 W kg^−1^	87% after 3000 cycles	[97]
Nb_18_W_16_O_93_	13 m LiAc	54 mAh g^−1^	41.9 Wh kg^−1^	20 kW kg^−1^	85% after 50,000 cycles	[82]
MnO_2_	21 m LiTFSI	303 F g^−1^	405 Wh kg^−1^	16.7 kW kg^−1^	90% after 3000 cycles	[98]
1T-MoS_2_	acetone and water added acetamide and lithium perchlorate	42.4 F g^−1^ at 1 A g^−1^	31.2 Wh kg^−1^	5.7 kW kg^−1^	91% after 20,000 cycles	[90]
Ti_3_C_2_T_x_	20 M LiCl	89.2 F cm^−3^	33.6 mWh cm^−3^	25 W cm^−3^	nearly no capacity decay after 10,000 cycles	[99]
Ti_3_C_2_ (anode) and MnO2 (cathode)	21 M potassium acetate	25 F cm^−3^	16.80 mWh cm^−3^	137 mW cm^−3^	93% after 10,000 cycles	[100]
Ti_3_C_2_T_x_	19.8 m LiCl	26 F g^−1^	9.2 Wh kg^−1^	41 W kg^−1^	Coulombic efficiency above 95%	[101]
PTCDI–rGO	32 m Ammonium acetate	165 mAh g^−1^	12.9 Wh kg^−1^	827 W kg^−1^	74% after 3000 cycles	[102]
PANI–rGO	N-methyl acetamide and lithium perchlorate in water and DMF	41.9 F g^−1^	28.2 Wh kg^−1^	5.6 kW kg^−1^	60% after 3000 cycles	[103]
VN//MnO_2_–AMSCs–GE	5 M LiTFSI	243 F g^−1^	21.6 mW cm^−3^	1539 mW cm^−3^	90% after 5000 cycles	[104]
rGO@ VO_2_	1 M LiPF_6_ in a 1:1 (*v*/*v*) ratio combining ethylene carbonate and diethyl carbonate	1214 mAh g^−1^	126.7 Wh kg^−1^	10,000 W kg^−1^	80% after 10,000 cycles	[105]
carbon nanostructures	LiOTf/NaOTf	284 F g^−1^	39.2 Wh kg^−1^	22 kW kg^−1^	85.5% after 10,000 cycles	[106]
PTCDI/rGO	DES (urea: magnesium chloride: lithium perchlorate: water)	76.5 mAh g^−1^ at 0.03 A g^−1^	53 Wh kg^−1^	1042 W kg^−1^	Coulombic efficiency is 99% after 200 cycles	[107]

## Data Availability

No data are available.
